# Evidence of Leaf Consumption Rate Decrease in Fall Armyworm, *Spodoptera frugiperda*, Larvae Parasitized by *Coccygidium* *luteum*

**DOI:** 10.3390/insects10110410

**Published:** 2019-11-16

**Authors:** Lakpo Koku Agboyi, Samuel Adjei Mensah, Victor Attuquaye Clottey, Patrick Beseh, Raymond Glikpo, Ivan Rwomushana, Roger Day, Marc Kenis

**Affiliations:** 1 Centre for Agriculture and Bioscience International (CABI), P.O. Box CT 8630, Cantonments, Accra GA 0376800, Ghana; samuelmensah1a@gmail.com (S.A.M.); v.clottey@cabi.org (V.A.C.); 2 Plant Protection and Regulatory Services Directorate (PPRSD), P.O. Box M37, Accra 00495426, Ghana; pkbeseh@gmail.com (P.B.); glikporaymond@yahoo.com (R.G.); 3 Centre for Agriculture and Bioscience International (CABI), 673 Limuru Road, Muthaiga, P.O. Box 633, Nairobi 00621, Kenya; i.rwomushana@cabi.org (I.R.); r.day@cabi.org (R.D.); 4 Centre for Agriculture and Bioscience International (CABI), 1 Rue des Grillons, 2800 Delémont, Switzerland; m.kenis@cabi.org

**Keywords:** maize, fall armyworm, *Spodoptera frugiperda*, biological control, endoparasitoid, *Coccygidium luteum*, feeding

## Abstract

Biological control is one of the best options for the sustainable management of the invasive maize pest *Spodoptera frugiperda* in Africa. However, there is limited knowledge of the efficacy of native natural enemies of *S. frugiperda* and their potential use in integrated pest management. The endoparasitoid wasp *Coccygidium luteum* is one of the natural enemies of *S. frugiperda* in Africa. This study assessed, under laboratory conditions, the effect of *C. luteum* on the leaf consumption rate of its host. Fifty first instar *S. frugiperda* larvae were exposed to *C. luteum* for oviposition and the maize leaf consumption rate of parasitized larvae was assessed and compared to 50 unparasitized larvae from the same cohort. *Coccygidium* *luteum* completed a generation, from egg to adult emergence, in 16.7 days. The leaf consumption rate of parasitized *S. frugiperda* larvae declined gradually compared to unparasitized larvae and the overall consumption reduction by parasitized *S. frugiperda* larvae was 89%. Our findings show that *C. luteum* could reduce damage caused by *S. frugiperda* to maize farms but, prior to its use in biological control programmes, further studies are needed to assess potential parasitism rates in field conditions and develop a cost-effective mass production system.

## 1. Introduction

Maize is a major staple food for millions of sub-Saharan Africans, and in Ghana it is ranked the first most important cereal crop [1]. However, the sustainable production of maize in Ghana has recently become constrained by the significant damage caused by the invasive fall armyworm, *Spodoptera frugiperda* (J.E. Smith) (Lepidoptera: Noctuidae), native to the Americas [2,3]. Since its first outbreak in West Africa in 2016, *S. frugiperda* had spread far and wide to all the sub-Saharan African countries, and to some parts of North Africa and Asia [4]. The rapid spread of *S. frugiperda* has been facilitated by its high dispersal ability and the wide range of host plants, including grasses and cereals [5,6,7,8,9,10,11,12]. The most hard-hit crop is largely maize, with significant yield and economic losses reported [9,13]. Environmental suitability modeling suggests that the pest is going to become resident in most places in Africa, making it a significant threat to food security, as maize is the staple food consumed by more than 300 million African smallholder households [2,14].

Emergency action against the pest in several African countries focused primarily on chemical control, a method widely promoted by some governments [15,16,17]. However, there is varied efficacy of the insecticides currently used in Africa [18], with a risk of insecticide resistance. Resistance to different commonly used pyrethroid, organophosphate and carbamate insecticides has been reported from some *S. frugiperda* populations in the Americas, especially Florida, from where the strains of the pest found in Africa are thought to have originated [11,19,20]. Besides, there is evidence that some commonly used pesticides against *S. frugiperda* remain in soil samples, with possible adverse effects on soil-borne organisms and other non-targeted species [21]. Therefore, an integrated pest management (IPM) approach for *S. frugiperda* is desirable. In Africa, the main IPM strategy being promoted is based on the judicious use of pesticides and lower risk methods, such as biopesticides, biological control and agronomic practices [22]. In relation to biological control, the major constraint in Africa is not only the limited knowledge on local natural enemies adapting to the pest, but also the capacity of exotic ones to adapt and be efficient under the African climatic conditions and agroecosystems. Given that in an IPM strategy, biological control is an important component [23], field surveys for natural enemies of *S. frugiperda* were conducted in Ghana in 2018. The survey identified *Coccygidium luteum* (Brullé) (Hymenoptera: Braconidae) as one of the major natural enemies of *S. frugiperda*, causing up to 19% parasitism in some parts of the country [24]. The species was also identified in Kenya and Tanzania as a common parasitoid of *S. frugiperda*, causing up to 9% parasitism [18]. *Coccygidium luteum* parasitizes different Lepidoptera species in Africa, such as *Condica capensis* (Guenée), *Crypsotidia mesosema* (Hampson), *Spodoptera exempta* (Walker), *Spodoptera exigua* (Hübner), *Prophantis* sp. and *Cydia ptychora* (Meyrick) [25].

*Coccygidium luteum* is widely distributed in Africa, as it has previously been recorded in Cameroon, Congo, the Democratic Republic of Congo, Ethiopia, Guinea, Kenya, Madagascar, Mauritius, Mozambique, Namibia, Niger, Nigeria, Rodrigues Island, Réunion, Senegal, Seychelles, Somalia, South Africa, Tanzania and Uganda [25]. It is a solitary koinobiont parasitoid belonging to the braconid sub-family Agathidinae, which contains more than 46 genera worldwide [26,27]. However, species in this sub-family are poorly studied [28], and little is known about their efficacy as biological control agents of insect pests. While a koinobiont parasitoid eventually kills its host, the benefit of biological control by such an agent can be undermined by the voracious feeding of the pest. Therefore, this study aims to assess the efficacy of *C. luteum* in reducing the leaf consumption rate of the *S. frugiperda* larvae on maize, and to understand the role this might play in the IPM of *S. frugiperda* in Ghana and other parts of Africa where the parasitoid is already present.

## 2. Materials and Methods 

### 2.1. Insect Collection and Rearing

Different instars of 154 larvae of *S. frugiperda* were collected from a maize farm in 2018, in Somanya in the Eastern region of Ghana (06.06225° N, 00.02358° W). The larvae were held in aerated plastic dishes (650 mL volume) containing a piece of tissue paper and were provided with fresh maize leaves collected in the maize farm, then transported to the biological control laboratory of the Plant Protection and Regulatory Services Directorate in Accra, Ghana. In the laboratory, the larvae were separated into individual cups (80 mL volume) and held under ambient conditions of temperature and relative humidity until the emergence of parasitoid adults or *S. frugiperda* moths. Adults of *C. luteum* and *S. frugiperda* that emerged were reared in separate aerated plastic cages measuring 18 dm^3^ and 50 dm^3^, respectively. Both insects were provided with 10% honey solution soaked in cotton wool, which was renewed every two days.

For the mass production of *S. frugiperda* larvae for experimentation, three- to four-week-old maize plants grown in a screenhouse without insecticide treatment were exposed to *S. frugiperda* moths in the aerated cage for 24 h. The plants were removed and replaced every 24 h and the egg masses laid were collected and kept separately in aerated plastic dishes (650 mL volume) containing a piece of tissue paper and maize leaves. Hatching larvae were provided with maize leaves until pupal stage. Some of the emerged female moths were paired with males in order to produce subsequent generations of the *S. frugiperda* larvae.

At each generation of *S. frugiperda*, five to 10 first instar larvae (L_1_) were exposed to each *C. luteum* female per day. The parasitized *S. frugiperda* larvae were removed and held in single cups as described above for the field collected larvae.

### 2.2. Feeding Tests on S. frugiperda Larvae

The mean temperature and relative humidity (RH) during this experiment were 32 ± 2 °C and 63 ± 2%, respectively, with a photoperiod of 12 h light:12 h dark. First instar larvae from the sixth generation of laboratory-reared *S. frugiperda* moths were introduced in aerated cages containing fifth generation *C. luteum* females mated 24 h prior to the experiment. A total of 50 one-day-old *S. frugiperda* L_1_ were exposed to seven *C. luteum* females. The behavior of *C. luteum* was observed to detect when parasitism occurred, i.e., when a female had inserted her ovipositor in the host larva. Each larva was removed immediately after being parasitized by the parasitoid and then held in a labeled single cup (80 mL volume). Another group of 50 *S. frugiperda* L_1_ belonging to the same cohort were held separately in single cups as a control. Leaves collected from three-week-old untreated screenhouse maize plants were used as the food source for the larvae over a period of 12 days, when pupation started to occur. The young tender leaves were cut into pieces measuring 30 cm^2^ and were provided to the parasitized and control larvae. Every 24 h, any remaining pieces of maize leaves that had not been consumed were removed from each cup containing either the parasitized or unparasitized *S. frugiperda* larvae. They were replaced immediately with fresh similarly sized pieces of leaves. In order to meet the food demand of unparasitized larvae, the number of pieces of maize leaf provided was increased as necessary from one to two pieces on the sixth to eighth day and from two to three pieces on the ninth to 12th day.

The pieces of maize leaves that remained after 24 h were collected daily from each cup, labeled and dried separately in an oven at 70 ± 10 °C for three days. At each renewal of feed, 40 pieces of maize leaves (30 cm^2^) were kept and dried in the same conditions to be used as a control. All the dried leaves were weighed using a precision laboratory analytical scale (COBOS ATY224 (Kyoto, Japan), Max 220 g, Min 10 mg, Precision (d): 0.1 mg). The daily consumption of maize by the *S. frugiperda* larvae was calculated as the difference in dry weight between the entire pieces of the maize leaves that were offered to the larvae and the non-consumed parts of the leaves left by the larvae in their cups.

### 2.3. Generation Time of C. luteum

The daily observations of the development of parasitized larvae provided data to calculate the generation time of *C. luteum* under laboratory conditions. The time from the day of oviposition to cocoon formation and adult emergence was measured for all individuals. 

### 2.4. Statistical Analysis

The mean dry weight of the pieces of maize leaves was used to calculate the food consumption of *S. frugiperda* larvae, without any transformation. The General Linear Model (GLM) with the repeated measures ANOVA procedure of SAS (version 9.4, Cary, NC, USA) was used to test the effect of the treatment and the time, as well as their interaction on the consumption of maize leaves by *S. frugiperda* larvae at the 0.05 alpha level.

## 3. Results

### 3.1. Generation Time of C. luteum

**Figure 1:**
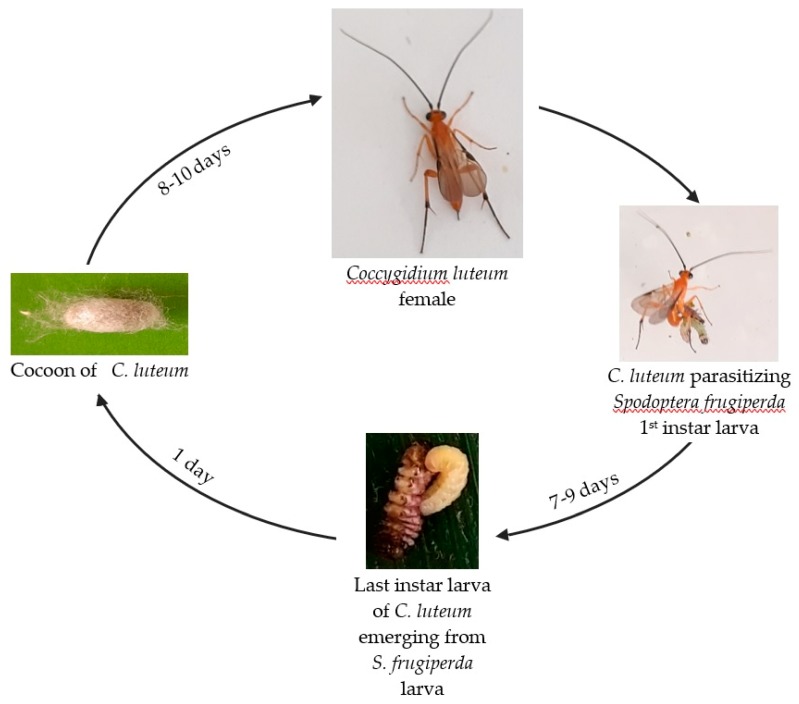
Generalized generation time of *Coccygidium luteum.*

From the 50 parasitized *S. frugiperda* larvae, 45 individuals of *C. luteum* completed their growth from egg to adult emergence and were used to assess the generation time of the parasitoid. The others reached the pupal stage but did not emerge. The female *C. luteum* wasp parasitized the exposed one-day-old L_1_ *S. frugiperda* by laying an egg inside its body. After hatching, the parasitoid larva fed inside the host, thereby affecting its growth. When the parasitoid larva completed its larval development, it emerged from the host (which caused its mortality) and built a white cocoon in which it pupated (Figure 1). The developmental period of the parasitoid from egg to pupal stage lasted eight to 10 days. The pupal stage also lasted eight to 10 days before the adult parasitoid emerged. The mean generation time of the 45 individuals having reached the adult stage was 16.71 ± 0.14 days (95% CI: 16.42–17.00). The minimum generation time was 16 days and the maximum 20 days.

### 3.2. Leaf Consumption Rate of Parasitized and Unparasitized S. frugiperda Larvae

**Figure 2:**
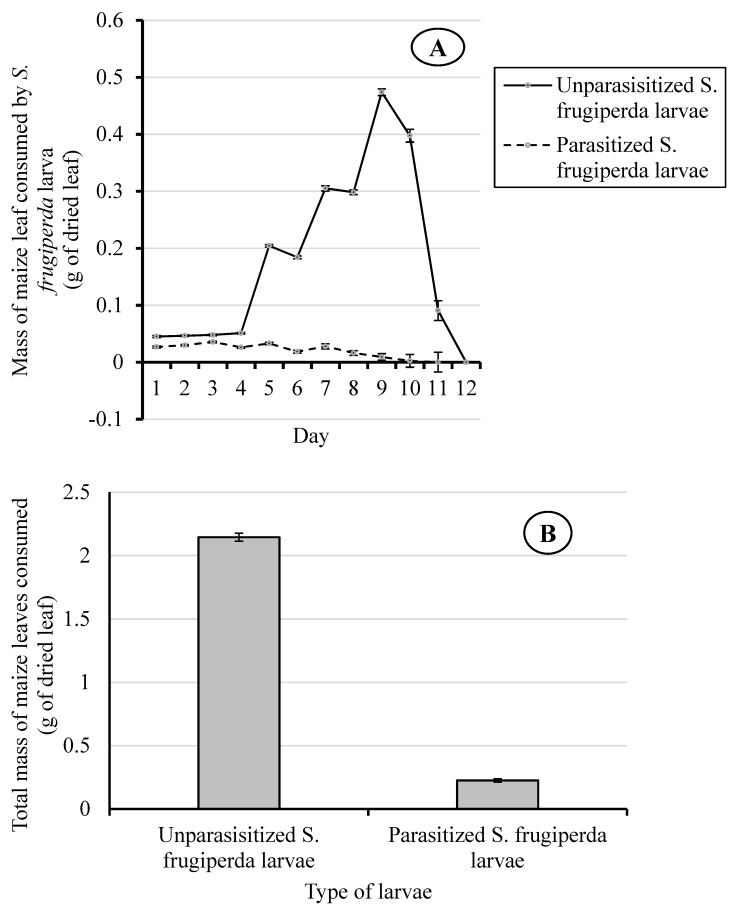
Daily mass of maize leaf consumed (**A**) and total consumption of maize leaves (**B**) by the parasitized and unparasitized *S. frugiperda* larvae.

The consumption rates of maize leaves by parasitized and unparasitized *S. frugiperda* larvae were significantly different (F_(1, 98)_ = 3155.01; *p* < 0.001) (Figure 2A) (Table S1). Moreover, they were significantly affected by time (Day) (F_(11, 1078)_ = 284.99; *p* < 0.001) and the interaction between treatment (larva status) and time (F_(11, 1078)_ = 310.38; *p* < 0.001) (Table S2).

From day one to four of the experiment, the daily dry weight of maize leaves consumed by unparasitized *S. frugiperda* larvae (0.045 ± 0.002 g to 0.051 ± 0.001 g dry wt. of leaf) was significantly higher (F_(1, 98)_ = 3155.01; *p* < 0.001) than the amount taken by the parasitized larvae (0.027 ± 0.002 g to 0.036 ± 0.002 g dry wt. of leaf) (Figure 2A). After day four, the leaf consumption by unparasitized *S. frugiperda* larvae increased quickly with time and it reached a peak on day nine (0.473 ± 0.006 g dry wt. of leaf) and then decreased until feeding ceased on day 12, as they went into pupation (Figure 2A). On day five, the consumption rate of the unparasitized *S. frugiperda* larvae went up sharply, consuming four times (0.204 ± 0.002 g dry wt. of leaf) the average daily mass of maize leaves they consumed during the first four days. 

In contrast, the rate of consumption of maize leaves by the parasitized larvae was relatively stable from day one to day seven, and then decreased to zero by day 10. On day nine, the leaf consumption by the parasitized larvae was estimated at 2% of that consumed by the unparasitized larvae (F_(1, 98)_ = 3155.01; *p* < 0.001) (Figure 2A). The total weight of maize leaves consumed during the larval stage by the parasitized *S. frugiperda* (0.226 g) was 11% of that consumed by unparasitized *S. frugiperda* (2.145 g) (Figure 2B).

## 4. Discussion

This study provides partial data on the life cycle of *C. luteum* on *S. frugiperda* and describes its ability to reduce the leaf consumption rate of its lepidopteran host. Even though the laboratory conditions probably accelerated the development of the parasitoid compared to field conditions, it appears that the parasitoid could produce several generations in 90 days, which corresponds to the development cycle of most early-maturing maize varieties in West Africa [29]. During the larval stage of *S. frugiperda*, the endoparasitoid decreased the host’s ability to feed on maize leaves and therefore its development. In total, feeding was reduced by 89% until the *S. frugiperda* larvae died. Other related studies have shown similar feeding reductions by koinobiont endoparasitoids in *Spodoptera* spp. For example, five parasitoids of *S. frugiperda*, namely *Aleiodes laphygmae* (Viereck)*, Campoletis sonorensis* (Cameron), *Chelonus insularis* (Cresson)*, Cotesia marginiventris* (Cresson) and *Meteorus laphygmae* (Viereck), decreased larval feeding by at least 80% [30]. This was also reported for the congeneric species *Spodoptera littoralis* (Boisduval), where food consumption and growth were significantly reduced by the endoparasitoids *Hyposoter didymator* (Thunberg) and *Chelonus inanitus* (Linnaeus) [31]. The effect of parasitism by *C. luteum* on *S. frugiperda* larvae could result from the different mechanisms associated with the parasitism process. Like all koinobiont endoparasitoids, *C. luteum* must overcome its host’s defense mechanisms to survive. In general, the defense mechanisms developed by koinobionts are passive and/or active. In the case of passive defense, females coat their eggs with a layer of protein to protect them against the host’s hemocytes or lay them in organs away from the circulation of hemocytes [32,33]. With an active defense mechanism, the endoparasitoid injects venom proteins or a virus during oviposition, in order to modify the behavior and damage the immune system of the host [34,35,36,37]. For example, another Braconidae, *Chelonus blackburni* (Cameron)*,* is able to decrease significantly the number of hemocytes in a parasitized *Helicoverpa armigera* (Hübner) larva and affect its midgut, thereby reducing the leaf consumption rate of the host and leading to death [23].

For *C. luteum*, a venom is likely to be involved in the parasitism mechanism, due to the rapid reduction of the host leaf consumption rate from the day the larva is parasitized. In addition, the female parasitoid struggles with the host when ovipositing, thereby provoking in the parasitized larva an elevation of its dopamine level, which plays an important role in the retardation of larval growth [38].

The reduction of leaf consumption rate is a common phenomenon for endoparasitoid wasps [39,40], however, a critical aspect is how it could help reduce the damage caused by an herbivorous host. The 89% reduction in the leaf consumption rate of *S. frugiperda* larvae from our study suggests that, if high parasitism levels could be achieved in the field, limited damage from *S. frugiperda* could occur on maize plants, even when the consumption rate is time-dependent after being parasitized.

The field parasitism data obtained from Ghana due to this parasitoid [24] suggests that *C. luteum* could be a promising candidate for the augmentative biological control of *S. frugiperda*. However, this requires unravelling more detailed knowledge on the parasitoids’ biology, such as the host stage and habitat specificity. It also requires the development of adapted and efficient mass rearing technologies. This is because the use of larval parasitoids as augmentative biological control agents is often hampered by prohibitive production costs. There are exceptions, such as the braconid *Habrobracon hebetor* (Say), which is used in several continents on various pests [41,42], but is produced cheaply on factitious hosts. It therefore remains to be seen whether *C. luteum* can be reared on hosts that can be mass produced at low cost. The cannibalistic behavior of *S. frugiperda* prevents its use in the mass rearing of larval parasitoids.

The abundance of *C. luteum* in Ghana also suggests the potential to be integrated in conservation biological control programmes. However, the environmental conditions, cropping systems and crop protection practices favoring the abundance of this and other parasitoids should be further investigated. This study has shown *C. luteum* to be able to parasitize early instar larvae, and therefore complement the action of other parasitoid species already present in Africa that target the egg stage or older larval stages [18,43,44].

Finally, efforts should be made at the national, regional and international level to promote the use of biological approaches for the management of invasive pests in African agriculture, which is often dominated by chemical control, including the use of highly restricted pesticides [17,45].

## 5. Conclusions

The results of this study are evidence that the endoparasitoid wasp, *C. luteum*, is able to reduce by up to 89% the leaf consumption rate of *S. frugiperda* on maize. Given that *C. luteum* is present in Western, Central, Eastern and Southern subregions of Africa, it could be considered as a potential parasitoid candidate for augmentative and conservation biocontrol strategies against *S. frugiperda*. The next steps are to assess whether an efficient and cost-effective rearing technique can be developed, and to carry out an assessment of environmental factors and cultural practices affecting field populations and the fitness of the parasitoid.

## Supplementary Materials

The following are available online at https://www.mdpi.com/2075-4450/10/11/410/s1. Table S1: Significant tests of hypotheses for between subjects (treatment: parasitized and unparasitized *S. frugiperda* larvae) effects, Table S2: Significant univariate tests of hypotheses for within subject effects.
