# Supplementary material for: Evidence of Leaf Consumption Rate Decrease in Fall Armyworm, Spodoptera frugiperda, Larvae Parasitized by Coccygidium luteum
Source: Insects. 2019 Nov 16;10(11):410. doi: 10.3390/insects10110410 (PMC6920753; doi:10.3390/insects10110410)
Supplement: Supplementary file 1 [file insects-10-00410-s001.pdf]

Table S1. Significant tests of hypotheses for between subjects (treatment: parasitized and unparasitized *S. frugiperda* larvae) effects.

**The GLM Procedure<br>Repeated Measures Analysis of Variance<br>Tests of Hypotheses for Between Subjects Effects**					
**Source**	**DF**	**Type III SS**	**Mean Square**	**F Value**	**Pr > F**
Treatment	1	7.67324309	7.67324309	3155.01	<0.0001
Error	98	0.23834392	0.00243208		

Analysis performed at the 0.05 alpha level.

Table S2. Significant univariate tests of hypotheses for within subject effects.

**The GLM Procedure<br>Repeated Measures Analysis of Variance<br>Univariate Tests of Hypotheses for Within Subject Effects**							
**Source**	**DF**	**Type III SS**	**Mean Square**	**F Value**	**Pr > F**	**G-G**	**H-F**
Day	11	6.61404123	0.60127648	284.99	<0.0001	<0.0001	<0.0001
Day*Treatment	11	7.20327241	0.65484295	310.38	<0.0001	<0.0001	<0.0001
Error (Day)	1078	2.27440943	0.00210984				

Greenhouse-Geisser Epsilon: 0.1995; Huynh-Feldt Epsilon: 0.2063; Analysis performed at the 0.05 alpha level.
